# Mutational analysis of severe acute respiratory syndrome coronavirus 2 in immunocompromised patients with persistent viral detection using whole genome sequencing

**DOI:** 10.1002/ctm2.1462

**Published:** 2023-11-06

**Authors:** Euijin Chang, Jungmin Lee, Jun‐Won Kim, Jong Hyeon Seok, Joon‐Yong Bae, Jeonghun Kim, Heedo Park, Choi‐Young Jang, Sung‐Woon Kang, So Yun Lim, Ji Yeun Kim, Jeong‐Sun Yang, Kyung‐Chang Kim, Joo‐Yeon Lee, Man‐Seong Park, Sung‐Han Kim

**Affiliations:** ^1^ Department of Infectious Diseases Asan Medical Center, University of Ulsan College of Medicine Seoul Republic of Korea; ^2^ Department of Microbiology Institute for Viral Diseases, Vaccine Innovation Center, Korea University College of Medicine Seoul Republic of Korea; ^3^ Division of Emerging Virus and Vector Research Center for Emerging Virus Research, National Institute of Infectious Diseases, Korea Disease Control and Prevention Agency Cheongju Republic of Korea

Dear Editor,

During the coronavirus disease 2019 (COVID‐19) pandemic of more than three years, several variants have evolved from the previously prevalent strains, being categorized as variants of concern (VOCs), variants of interest (VOIs), variants of high consequence and variants being monitored.^1^ The origin of new severe acute respiratory syndrome coronavirus 2 (SARS‐CoV‐2) variants is unclear, but one possible explanation is that they stem from immunocompromised patients.^2^ Whole‐genome sequencing (WGS) is a useful tool for detecting new mutations and emerging SARS‐CoV‐2 variants.^3^ Here, we used WGS to investigate the features of nonsynonymous SARS‐CoV‐2 mutations that appeared in immunocompromised patients with persistent viral detection during the Omicron‐prevalent era.

This prospective study was conducted at a 2732‐bed tertiary teaching hospital from February to November 2022. We enrolled immunocompromised adults within 12 weeks of initial SARS‐CoV‐2 diagnosis and gathered nasopharyngeal swabs, saliva and blood samples on a weekly basis. We also performed real‐time reverse transcription‐polymerase chain reaction tests for SARS‐CoV‐2, viral cultures, plaque reduction neutralization tests, and WGS on at least two serial samples from each patient. The details of patient enrollment, sample collection, and laboratory procedures are explained in the Supporting Information.

A total of 37 WGS results from 13 SARS‐CoV‐2 patients were included in the final analysis (Figure S1). The baseline features of the study subjects are described in Table S1. The WGS analysis results were obtained from each immunocompromised patient with a median frequency of three times (interquartile range [IQR] 2–3). The median interval between consecutive WGS analyses was 20 days (IQR 15–46 days). The patients acquired a median of two nonsynonymous mutations (IQR 1–7), excluding temporary mutations. The specific mutations compared with the Wuhan Hu‐1 reference genome and the acquired mutations in subsequent WGS are presented for each patient in Figure 1 and Table 1.

FIGURE 1Nonsynonymous mutations of severe acute respiratory syndrome coronavirus 2 (SARS‐CoV‐2) acquired by each patient. Each alphabet character represents the nonsynonymous mutations compared with the Wuhan‐Hu‐1 reference genome. The black boxes present the newly acquired nonsynonymous mutations compared with the initial SARS‐CoV‐2 genome in each patient. The day on which each specimen was collected is indicated to the left normed to the diagnosis day (D0). The results of the SARS‐CoV‐2 culture are displayed using red symbols: (+) for positive and (‐) for negative. All nonsynonymous mutations were distributed throughout the entire SARS‐CoV‐2 genome, and each patient acquired unique and different mutations.

**Figure:**
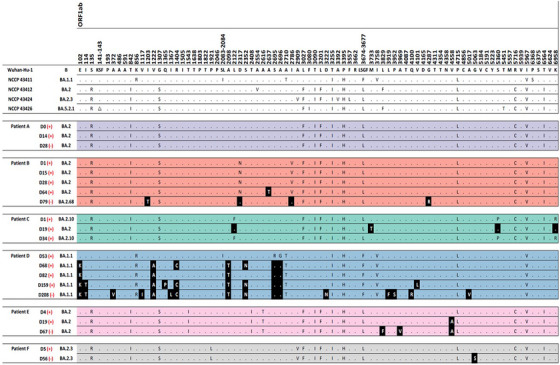

**Figure:**
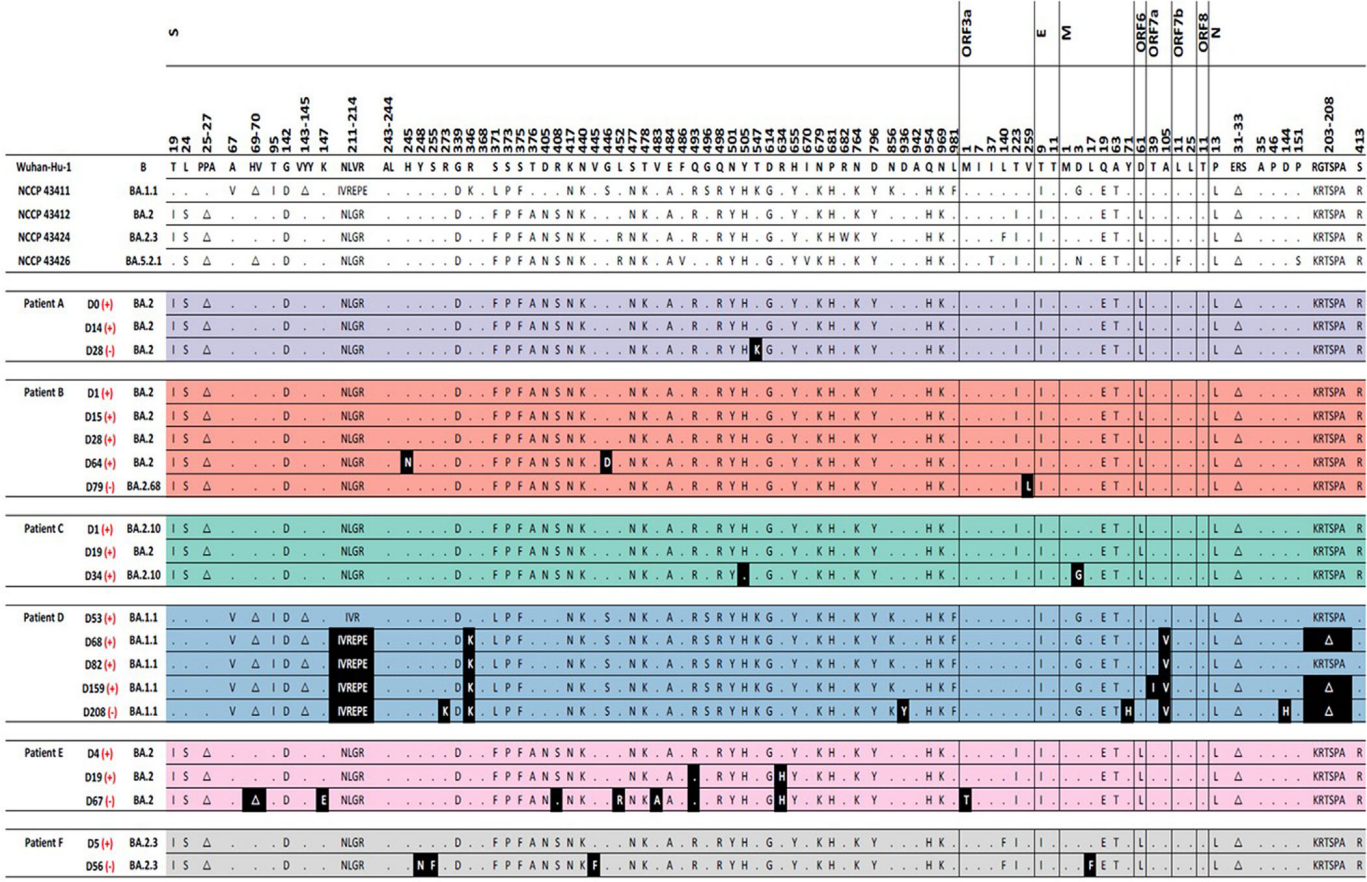

**Figure:**
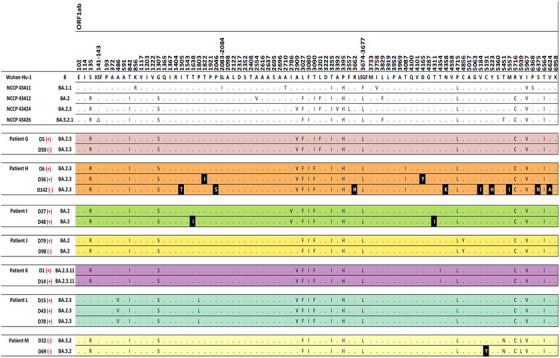

**Figure:**
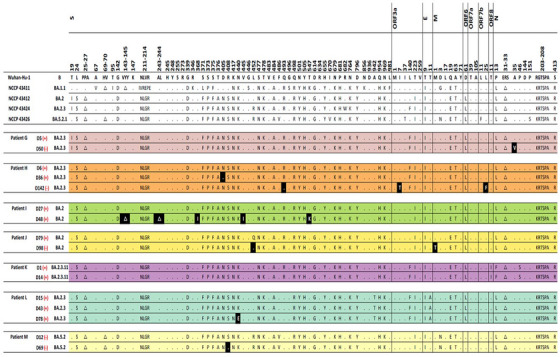

**TABLE 1:** Acquired nonsynonymous mutations in the severe acute respiratory syndrome coronavirus 2 (SARS‐CoV‐2) genomes of immunocompromised patients.

		Acquired mutation		
Patient	Initial lineage	**Persistent**	**Temporary**	**Undetermined**
A	BA.2			S:T547K (D28)
B	BA.2		ORF1ab:A2637T (D64)	ORF1ab:I1203T (D79)
			S:H245N (D64)	ORF1ab:N2317D (D79)
			S:G446D (D64)	ORF1ab:V2786I (D79)
				ORF1ab:G4287R (D79)
				ORF3:V259L (D79)
C	BA.2.10		ORF1ab:F2122L (D19)	M:D3G (D34)
			ORF1ab:M3733T (D19)	S:H505Y (D34)
			ORF1ab:P5360S (D19)	
			ORF1ab:R6958K (D19)	
D	BA.1.1	ORF1ab:E102K (D68)	ORF1ab:Q1365P (D159)	ORF1ab:A372V (D208)
		ORF1ab:I114T (D159)	ORF1ab:V4101L (D159)	ORF1ab:V1117I (D208)
		ORF1ab:V1222A (D68)	ORF7:T39I (D159)	ORF1ab:I1367L (D208)
		ORF1ab:R1404C (D159)		ORF1ab:D3222N (D208)
		ORF1ab:A2098T (D68)		ORF1ab:L3919F (D208)
		ORF1ab:S2352N (D159)		ORF1ab:P3952S (D208)
		ORF1ab:R2695S (D68)		ORF1ab:Q4100R (D208)
		ORF1ab:G2696A (D68)		ORF1ab:A5017V (D208)
		S :I210V (D68)		S:R273K (D208)
		S :V213E (D68)		S:D936Y (D208)
		S:R346K (D68)		M:Y71H (D208)
		ORF7:A105V (D68)		N:D144H (D208)
		N:KRTSPA203‐208Del (D159)		
E	BA.2	ORF1ab:V4558A (D19)		ORF1ab:L3829F (D67)
		S:R493Q (D19)		ORF1ab:A3969V (D67)
		S:R634H (D19)		S:HV69‐70Del (D67)
				S:K147E (D67)
				S:S408R (D67)
				S:L452R (D67)
				S:V483A (D67)
				ORF3:M1T (D67)
F	BA.2.3			ORF1ab:G5063S (D56)
				S:Y248N (D56)
				S:S255F (D56)
				S:V445F (D56)
				M:L17F (D56)
G	BA.2.3			N:A35V (D50)
H	BA.2.3		ORF1ab:T1822I (D36)	ORF1ab:I1505T (D142)
			ORF1ab:D4165Y (D36)	ORF1ab:P2046S (D142)
			S :N405D (D36)	ORF1ab:R3662H (D142)
				ORF1ab:N4358K (D142)
				ORF1ab:V5184I (D142)
				ORF1ab:Y5223H (D142)
				ORF1ab:M5557I (D142)
				ORF1ab:S6375N (D142)
				ORF1ab:V6624A (D142)
				S:R493Q (D142)
				ORF3:I7T (D142)
				ORF7b:L25F (D142)
I	BA.2			ORF1ab:T1638I (D48)
				ORF1ab:T4311I (D48)
				S:Y144Del (D48)
				S :AL243‐244Del (D48)
				S:L368I (D48)
				S:V445I (D48)
				S:T547K (D48)
J	BA.2			S:Q452L (D98)
				M:M1T (D98)
K	BA.2.3.11			
L	BA.2.3			S:K440E (D78)
M	BA.5.2			ORF1ab:C5191Y (D69)
				S:S408R (D69)

Abbreviation: D, day.

Each mutation is presented with the day it was first detected in the respiratory specimen, relative to the diagnosis day (D0). Mutations in the underlined bold text represent those reported to be associated with immune evasion or other SARS‐CoV‐2 major variants.

Among the total 87 nonsynonymous mutations, 16 (18.4%) and 13 (14.9%) mutations were classified as persistent and temporary mutations, respectively. More than half of the mutations were detected in the ORF1ab region (Figure S2). There were 29 mutations in the S region, 12 of which were associated with immune evasion (see Supporting Information). Also, 13 mutations in the ORF1ab, S, and M regions were the defining mutations of the major variants, including Omicron BA.1, BA.2.75, BA.4/5, and several XBB subvariants,^4^ and eleven of these mutations occurred in the S region (Table 2). The proportion of acquired mutations that were defining mutations of other variants was higher in the S region (11/29, 37.9%) than in the whole genomic region (13/87, 14.9%). Table S2 outlines the number of nonsynonymous mutations associated with immune evasion and the defining mutations of the major variants for each patient.

**TABLE 2:** Acquired nonsynonymous mutations that are defining mutations of other major variants.

Non‐synonymous mutation	Associated major variants
ORF1ab:L3829F	BQ.1, XBB.1.16
S:HV69‐70Del	B.1.1.7 (Alpha), B.1.525 (Eta), BA.1, BA.4, BA.5, BQ.1
S:Y144Del	B.1.1.7 (Alpha), B.1.525 (Eta), BA.1, XBB, XBB.1.5, XBB.1.16
S:K147E	BA.2.75
S:I210V	BA.2.75
S:V213E	XBB, XBB.1.5, XBB.1.16
S:L368I	XBB, XBB.1.5, XBB.1.16
S:L452Q	BA.2.12.1, C.37 (Lambda)
S:L452R	BA.4, BA.5, BQ.1, B.1.617.1 (Kappa), B.1.427/B.1.429 (Epsilon), B.1.617.2 (Delta)
S:R493Q	BA.2.75, BQ.1, XBB, XBB.1.5, XBB.1.16
S:Y505H	BA.4, BA.5, BA.2.12.1, BA.2.75, BQ.1, XBB, XBB.1.5, XBB.1.16
S:T547K	BA.1
M:D3G	BA.1

The V792I mutation in the *nsp12*, also known as V5184I in the ORF1ab region, is reported to be associated with viral resistance to remdesivir.^5^ Patient H acquired this mutation 142 days after SARS‐CoV‐2 diagnosis. Before the acquisition of this mutation, the patient had prolonged exposures to remdesivir, dexamethasone, and baricitinib for 28, 17 and 15 days, respectively (Figure 2). This patient also received high‐dose steroids (≥ equivalent doses of prednisolone 0.3 mg/kg daily) for more than two months.

FIGURE 2Changes of severe acute respiratory syndrome coronavirus 2 (SARS‐CoV‐2) viral loads, titers of neutralizing antibodies, and nonsynonymous mutations in sequenced SARS‐CoV‐2 genomes in each patient over time and the coronavirus disease 2019 (COVID‐19) treatments used. Red and blue lines represent the changes in the amounts of genomic RNA and the 50% neutralization doses of neutralizing antibodies against SARS‐CoV‐2, respectively. Filled and empty circles on the red lines indicate the results of SARS‐CoV‐2 culture. Asterisks mark the days when whole‐genome sequencing was conducted, with nonsynonymous mutation detection highlighted in yellow boxes. The identified SARS‐CoV‐2 lineages for each patient are displayed in the upper left olive‐coloured boxes. Additionally, the time points and durations of treatments, including remdesivir, dexamethasone, baricitinib, tocilizumab, and evusheld, are indicated in each graph.

**Figure:**
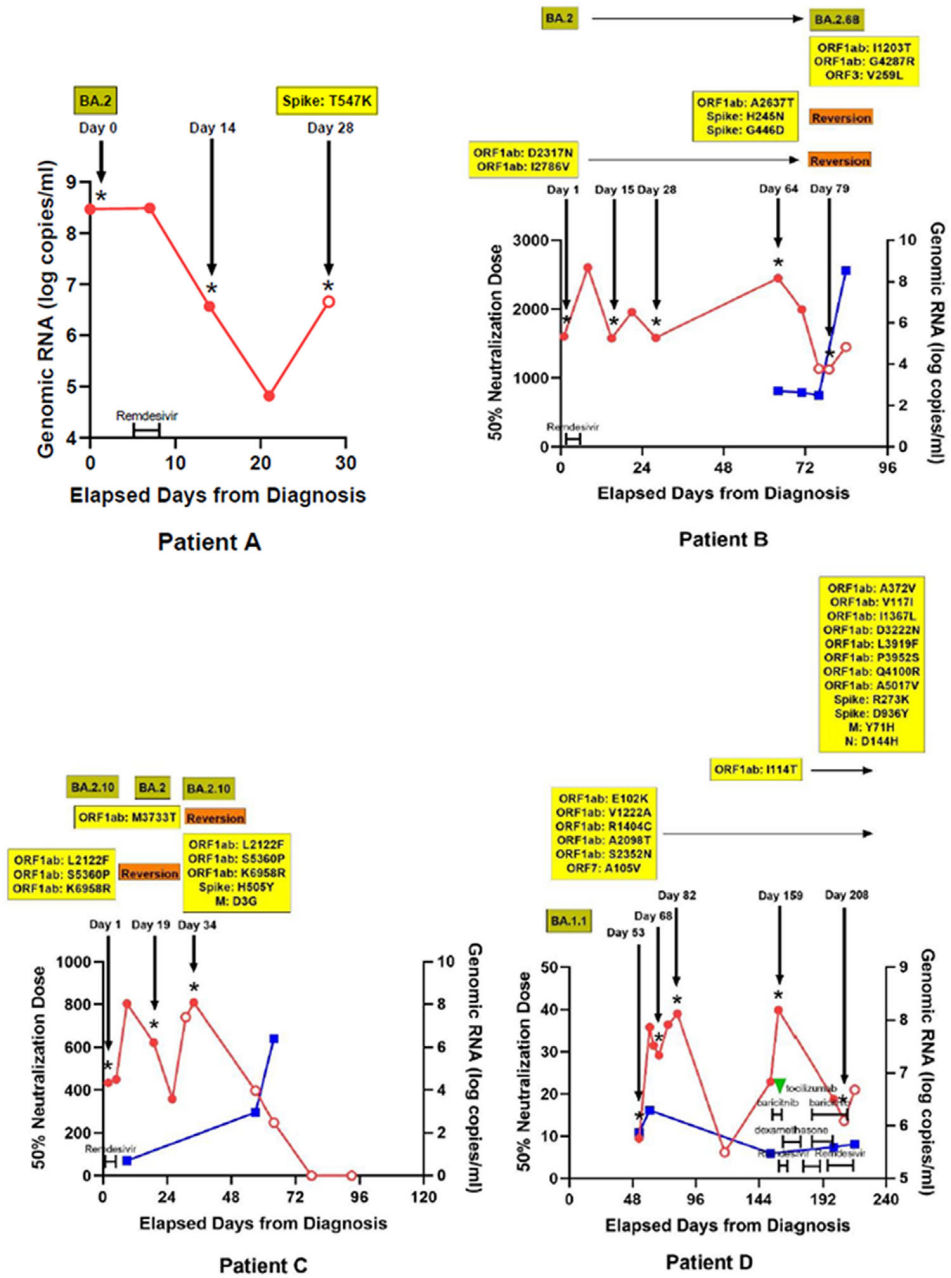

**Figure:**
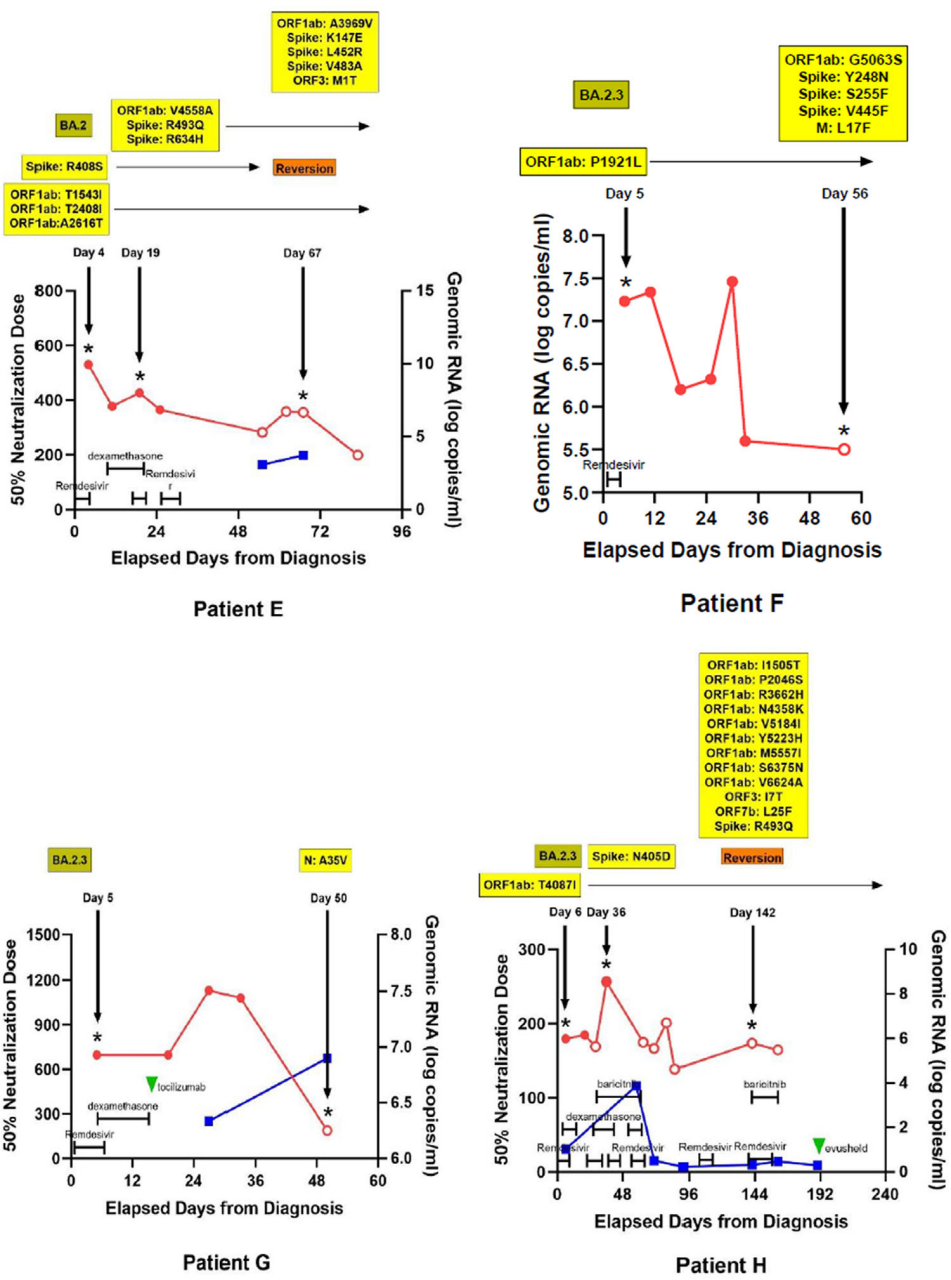

**Figure:**
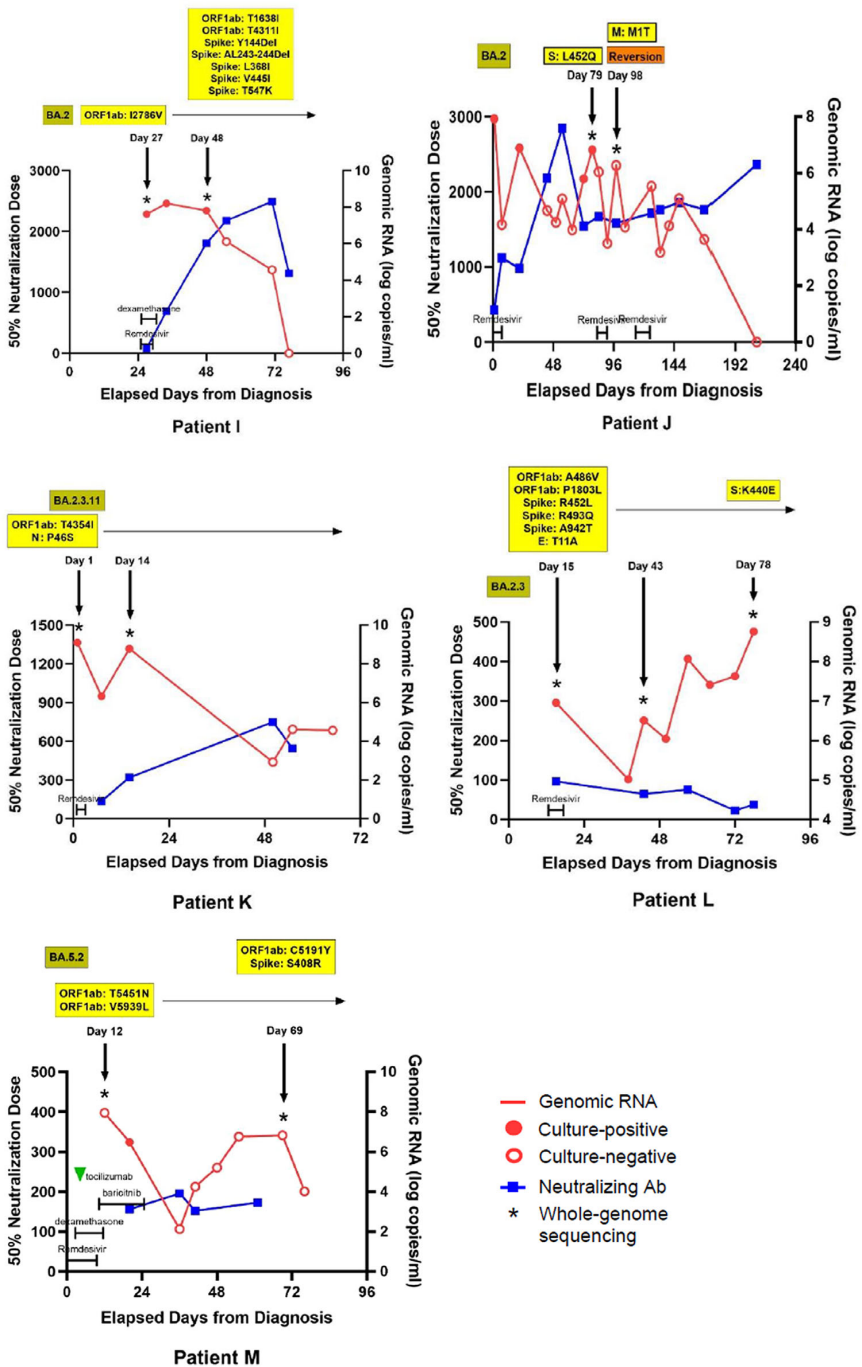

While B‐cell depletion is considered the main factor affecting the period of SARS‐CoV‐2 shedding,^6^ the presence of high neutralizing antibody titers does not always ensure eradication of SARS‐CoV‐2 infection.^7^ Patient J shed the virus persistently from days 72–79 despite maintaining a high titer of neutralizing antibodies since the initial COVID‐19 diagnosis (Figure 2). WGS analyses were conducted on days 79 and 98, and the missense mutation S:L452Q, detected on day 79, seemed to have ‘reverted’ by day 98. This mutation has been reported to be associated with immune evasion and a decreased sensitivity to neutralizing antibodies.^8^ While we could not determine the exact duration of the presence of the S:L452Q mutation or assess the status of T‐cell immunity for this patient, persistent viral shedding might be attributed to this mutation and its diminished sensitivity to neutralizing antibodies.

This study investigated the dynamics and characteristics of SARS‐CoV‐2 mutations in immunocompromised patients with persistent viral detection during the Omicron era. Each patient acquired a median of two amino acid substitutions over a median of 51 days, which equals 14.2 substitutions per year. In comparison, other studies from the pre‐Omicron era reported nonsynonymous mutation rates of 24.4–52.4 substitutions per year for immunocompromised patients.^7^, ^9^, ^10^ While our study involved a larger cohort of such patients, some exhibited a milder immunocompromised status than those in previous studies, potentially leading to variations in the mutation rates.

Several mutations seem to have emerged sporadically, distributed throughout the whole SARS‐CoV‐2 genome. This distribution pattern mirrors findings from an earlier study that also reported a sporadic distribution of various mutations across the SARS‐CoV‐2 genome in immunocompromised individuals.^2^, ^7^, ^9^, ^10^ The ORF1ab region, accounting for up to 21,290 nucleotides (71.2%) of the 29,900 total, housed more than half of the nonsynonymous mutations identified in this study. The S region, consisting of 3,822 nucleotides (12.8%), harboured about one‐third of the mutations. The adjusted mutation numbers per kilobase were 2.1 for the ORF1ab region and 7.6 for the S region. Additionally, mutations known to contribute to immune escape, or those defining other variants designated as VOIs or VOCs, primarily arose in the S region. This observation aligns with findings from other studies.^7^, ^9^

The immunocompromised patients in this study were predominantly infected with BA.2 or BA.2.3 sub‐lineages. We identified mutations typical of BA.4/5, BA.2.75, BQ.1 and various XBB subvariants in the SARS‐CoV‐2 genomes from these patients. Notably, during the pre‐Omicron era, immunosuppressed patients were found to acquire nonsynonymous mutations linked to subsequent SARS‐CoV‐2 lineages.^2^, ^7^, ^9^ These observations suggest that persistent viral infections in immunocompromised patients could drive the acquisition of new mutations, leading to the adaptive evolution of SARS‐CoV‐2. Therefore, tracking these mutations might provide insights into viral adaptation and the advent of new SARS‐CoV‐2 variants.

This study has several limitations. Viral evolution within populations can be influenced by infection prevalence and immune landscapes, as well as ethnic genetic predispositions.^11^ Our study was conducted during the Omicron‐prevalent era, and the lack of data from the pre‐Omicron period limits the generalization of our findings. Also, the patients in our study might not fully represent the spectrum of immunity statuses in immunocompromised patients. Specifically, eleven out of the thirteen patients in our study had hematologic malignancies, and eight had not received SARS‐CoV‐2 vaccines. This particular immunity profile could give rise to mutations distinct from those observed in other immunocompromised populations.^11^ Consequently, there might be some potential for regional or immunological biases in our findings.

In conclusion, during the Omicron‐prevalent era, SARS‐CoV‐2 genomes of immunocompromised individuals with persistent viral detection exhibited several mutations. These mutations have been reported to be associated with immune evasion, remdesivir resistance and new variant emergence. Given the rise of new subvariants with mutations associated with immune evasion or remdesivir resistance and the potential for immunocompromised individuals to shed viable viruses, decisions regarding the termination of isolation for immunocompromised patients with SARS‐CoV‐2 infection should be approached with caution.

## CONFLICT OF INTEREST STATEMENT

The authors declare no conflict of interest.

## FUNDING INFORMATION

Korea National Institute of Health, Grant/Award Numbers: 2022‐ER1609‐00, 2022‐NI‐043‐00 and 6634‐325‐210; Ministry of Science and Information & Communications Technology, Republic of Korea, Grant/Award Number: NRF‐2022M3A9I2017241; Ministry of Education, Republic of Korea, Grant/Award Number: 2021R1A6C101C570

## Supporting information

Supporting information

Supporting information

Supporting information

Supporting information

Supporting information

Supporting information

## Data Availability

All data supporting the findings of this study are available within the paper and its supplementary material and from the corresponding authors upon reasonable request.
