# Supplementary material for: Mutational analysis of severe acute respiratory syndrome coronavirus 2 in immunocompromised patients with persistent viral detection using whole genome sequencing
Source: Clin Transl Med. 2023 Nov 6;13(11):e1462. doi: 10.1002/ctm2.1462 (PMC10626488; doi:10.1002/ctm2.1462)
Supplement: Supplementary file 1 — Supporting information [file CTM2-13-e1462-s004.docx]

**SUPPORTING INFORMATION**

**MATERIALS AND METHODS**

**Study participants and specimen collection**

Following the definition of “immunocompromised” by the Centers for Disease Control and Prevention,^1^ we enrolled adults (age ≥ 18 years) with hematologic malignancies or those who had received solid organ transplants. All participants were within 12 weeks of their initial diagnosis of SARS-CoV-2 infection. All of the patients were confirmed to have SARS-CoV-2 infections by nasopharyngeal swab polymerase chain reaction (PCR), and patients with a history of prior SARS-CoV-2 infection were excluded.

We obtained nasopharyngeal swabs, saliva, and blood samples weekly from the enrolled patients. SARS-CoV-2 loads were measured in each saliva sample and nasopharyngeal swab by genomic RNA real-time reverse transcription-PCR (RT-PCR), and those with positive results were cultured to detect persistent viral shedding. Also, at least two or more respiratory specimens with a cycle threshold value of less than 30 were selected for SARS-CoV-2 whole-genome sequencing (WGS) for each patient. Blood samples were used to measure neutralizing antibodies against SARS-CoV-2 Omicron variants.

Clinical information about the study participants, including age, sex, comorbidities, history of vaccination against SARS-CoV-2, and treatments for SARS-CoV-2 infection was reviewed in their electronic medical records, and written informed consent was obtained from the participants. All of the participants agreed that their genomic and laboratory data could be analyzed and publicized without disclosing personal information. This study was approved by the internal review boards of the Asan Medical Center (IRB-2022-1054).

**Measurement of SARS-CoV-2 RNA**

PCR reaction mixtures (20 μL) contained 5 μL of extracted RNA or in vitro-synthesized control RNA, 500 and 200 nM of S gene primers and probes, 500 and 250 nM of N gene primers and probes, 250 and 125 nM of internal control primers and probes, respectively, 0.1 μL of 200× enzyme mix, and 4 μL of 5× master mix (LightCycler Multiplex RNA Virus Master, Roche, Basel, Switzerland) (Appendix Table 1).

RNA amplification was performed using a LightCycler 96 system (Roche) as follows: (i) reverse transcription at 50℃ for 10 minutes → (ii) initial denaturation at 95℃ for 5 minutes → (iii) 45 cycles of 2-step amplification → (iv) denaturation at 95℃ for 10 seconds → (v) annealing and elongation at 60℃ for 30 seconds → (vi) final extension at 60℃ for 5 minutes.

We generated calibration curves by conducting six independent assays with samples of synthetic control RNA serially diluted from 5$\times$10^7^ to 5$\times$10^1^ copies/μL. The limit of detection in this assay was 5 copies/reaction (2.6 log copies/ml of specimen), and viral copy numbers were calculated by plotting Ct values of the SARS-CoV-2 N gene against log copies/reaction.^2^

**Culture of SARS-CoV-2**

Respiratory specimens with positive SARS-CoV-2 RNA RT-PCR results were cultured to detect viable viruses in a biosafety level 3 (BSL3) facility. The culture method for SARS-CoV-2 followed previously described protocols.^3,4^ Samples were inoculated into prepared Vero-E6 cells (1.5×10^5^ cells/well). The cells were incubated for 1 hour at 37℃ with 5% CO_2_. After removal of the inoculum, the cells were further incubated with Dulbecco’s modified Eagle’s medium containing 2% fetal bovine serum and 1% penicillin-streptomycin.

For one week, we monitored the infected cells for cytopathic effects. On the seventh day, supernatants from the infected cells were collected and the SARS-CoV-2 viral load was measured by real-time RT-PCR. A culture was considered positive when cytopathic effects were observed in the inoculated cells and SARS-CoV-2 viral loads in the supernatants exceeded 10^6^ copies/ml.

If the viral loads were below 10^6^ copies/ml in supernatants from cells showing cytopathic effects, the culture tests were repeated. The supernatants were reintroduced into other cells and the incubation was repeated as described above.

To confirm virus viability, real-time RT-PCR of SARS-CoV-2 was conducted using supernatants from these sub-passaged cells. A positive viral culture was confirmed if cytopathic effects were detected in the sub-passaged cells and SARS-CoV-2 RNA was present in the supernatants.

**WGS and mutational analysis**

Two or more respiratory samples, taken with an interval of at least two weeks and with Ct values below 30, were chosen from each patient for WGS. Viral nucleic acids were extracted using the automatic extraction instrument (Maxwell RSC 48 system, Promega, Madison, WI, USA) at the Vaccine Innovation Center of Korea University. To isolate pure SARS-CoV-2 RNA, human RNA was removed from samples with a NEBNext rRNA Depletion Kit (New England Biolabs, Ipswich, MA, USA). Libraries were prepared with a TruSeq RNA sample preparation kit v2 (Illumina, San Diego, CA, USA). The enriched libraries were quantified using a Kapa Library Quantification Kit (Roche, Basel, Switzerland) and sequenced with a Miseq reagent kit v2 (300 cycles) (Illumina). Sequences were analyzed on a CLC Genomics Workbench 10 (QIAGEN).

FASTQ files containing the raw reads were trimmed and mapped to the reference Wuhan-Hu-1 sequence (GeneBank accession number NC_045512.2). Viral genome assembly and variant calling for each genome were done with Multiple Alignments using Fast Fourier Transform (MAFFT), and pangolin lineages were identified with the Pangolin software, version 4.2 (<https://pangolin.cog-uk.io/>).

We identified the most frequent single nucleotide polymorphisms leading to new nonsynonymous mutations by comparing them to the SARS-CoV-2 genome from each patient's initial sample. Then, we investigated whether these identified mutations were associated with immune evasion or resistance to remdesivir, referencing earlier reports and studies. We also checked if these mutations were included in the defining mutations of the major SARS-CoV-2 variants.^5^ Based on the results of the WGS analyses, we categorized the acquired nonsynonymous mutations into three groups: (i) persistent mutations detected in at least two consecutive samples; (ii) temporary mutations that appeared only once and disappeared from subsequent samples; and (iii) undetermined mutations that occurred only in a patient’s last sample, leaving their persistence unclear.^6^

**Plaque reduction neutralization test (PRNT)**

Neutralizing antibodies in blood were measured in a BSL3 facility. The live SARS-CoV-2 viruses used in this assay were Omicron variants BA.1 (hCoV-19/Korea/NCCP 43408/2021, EPI_ISL_2887353), BA.2 (hCoV-19/Korea/NCCP 43412/2022, EPI_ISL_13086512), and BA.5 (hCoV-19/Korea/NCCP 43426/2022, EPI_ISL_13086516) provided by the Korean Disease Control and Prevention Agency. When a patient was infected with the Omicron BA.1 lineage, the PRNT was conducted against Omicron BA.1, and similarly for the Omicron BA.2 and BA.5 lineages.

PRNT was performed as in previously described methods.^7,8^ Serially diluted serum and an equal volume of virus (40 plaque-forming units per well) were mixed and incubated at 37℃ for two hours. Next, this mixture was inoculated into a 24-well plate with Vero E6 cells ($1\times{10}^{5}$ cells/well) and incubated at 37℃ for one hour. Then, 1 ml of 0.5% agarose (Lonza) was added to each well. After two to three days of incubation, the cells were fixed with 4% paraformaldehyde and stained with crystal violet to visualize the plaques. Using the Spearman-Karber formula,^9^ the 50% neutralization dose (ND50) of antibodies against SARS-CoV-2 was calculated, which resulted in a 50% reduction of plaques, and it is presented as the reciprocal of serum dilution.

**Statistical analysis**

Fisher’s exact test was used to analyze categorical variables and the Mann-Whitney U test or Student’s t-test was performed for continuous variables, depending on whether the data were normally distributed or not. Two-tailed *p*-values less than 0.05 were considered statistically significant. We used R version 4.2.3 (R Foundation for Statistical Computing, Vienna, Austria) and GraphPad Prism version 9.0 (GraphPad Software, San Diego, California) to analyze and present the results.

**EXTENDED RESULTS**

**Characteristics of acquired mutations associated with immune evasion**

Of the 28 nonsynonymous mutations observed in the S region of SARS-CoV-2 genomes, 12 have previously been reported to be associated with immune evasion. The S:G446D mutation, located in the receptor-binding domain (RBD), exhibits significantly reduced susceptibility to bebtelovimab and other monoclonal antibodies against SARS-CoV-2.^10,11^ S:D405N reduces the effectiveness of sarbecovirus-neutralizing antibodies, and is a defining mutation for omicron variants such as BA.2, BA.2.12.1, BA.2.75, BA.4, BA.5, BQ.1, XBB, and XBB.1.5.^12^ The S:K147E mutation, characteristic of the BA.2.75 variant, is situated in the N-terminal domain of the S region. It confers resistance to both polyclonal sera and monoclonal antibodies, including bebtelovimab.^13^ S:R408S is the defining mutation of several Omicron subvariants, such as BA.2, BA.2.12.1, BA.2.75, BA.4, BA.5, BQ.1, XBB, and XBB.1.5. It may alter the conformation of the RBD, resulting in reduced susceptibility to monoclonal antibodies.^14^ S:L452R, unique to Omicron subvariants BA.4, BA.5, and BQ.1, resides in the RBD and offers escape from human leukocyte antigen-restricted cellular immunity.^15^ S:V483A, located in the receptor-binding motif, diminishes viral susceptibility to certain monoclonal antibodies, such as bamlanivimab.^16^ S:V445F, another RBD mutation, has been detected in other immunocompromised patients^17^ and is known to resist certain monoclonal antibodies.^18^ The mutations S:HV69-70Del, S:Y144Del, and S:AL243-244Del appear in the recurrent deletion region of the Spike tip and offer resistance to some monoclonal antibodies,^19^ while S:H505Y is associated with evasion from BA.1-specific neutralizing antibodies.^12^ Lastly, S:L452Q, specific to the Lambda variant and found in the RBD, diminishes susceptibility to convalescent plasma and vaccines.^20^ It is also the defining mutation of the BA.2.12.1 Omicron subvariant. Notably, a booster vaccination with Comirnaty^®^ (Pfizer Inc., Manhattan, New York, United States) against SARS-CoV-2 produces a less effective neutralizing antibody response to BA.2.12.1 compared to BA.1 or BA.2.^21^

**Probability of each mutation being found in the SARS-CoV-2 genomic database**

We retrieved SARS-CoV-2 sequences from the GISAID EpiCoV database using filters with "Low coverage excluded," "Complete," and "High coverage."^22,23^ From this, we obtained 62,219 sequences for BA.2, 6,006 for BA.2.3, 656 for BA.5.2, and 1,721 for BA.1.1. Upon aligning these sequences, we examined the prevalence of each mutation found in the immunocompromised patients.

Of the 87 nonsynonymous mutations identified, frequencies of 85 mutations being observed in the retrieved SARS-CoV-2 genomic sequences were less than 1% (Appendix Table 2). Among them, 38 were uniquely found in our study, and 25 (65.8%) of these were in the ORF1ab region.^24,25^ In the ORF1ab region, 25 (55.6%) out of 45 mutations were exclusively detected in our study. In the Spike region, only seven (24.1%) out of 29 mutations were solely found in our study.

**Hotspot mutations found within the SARS-CoV-2 genome from the study patients**

Previous studies have identified numerous hotspot mutations within the SARS-CoV-2 genome, including S:HV69-70Del, S:Y144Del, and S:L452R, which have emerged with higher frequencies than others.^26,27^ In this study, patient E acquired two hotspot mutations, S:HV69-70Del and S:L452R, and patient I acquired one hotspot mutation, S:Y144Del. These three hotspot mutations are the defining mutations of other major SARS-CoV-2 variants.^5^ While the mutations S:S408R, S:T547K, and S:R493Q were observed in more than one patient in our study, none had been previously reported as hotspot mutations. However, both S:T547K and S:R493Q are defining mutations of Omicron subvariants.^5^

**REFERENCES**

1. Centers for Disease Control and Prevention. “People who are immunocompromised.” Accessed April 8, 2023. https://www.cdc.gov/coronavirus/2019-ncov/need-extra-precautions/people-who-are-immunocompromised.html

2. Han MS, Byun JH, Cho Y, Rim JH. RT-PCR for SARS-CoV-2: quantitative versus qualitative. *Lancet Infect Dis*. 2021;21:165. doi:https://doi.org/10.1016/S1473-3099(20)30424-2

3. Kang SW, Kim JW, Kim JY, et al. Characteristics and risk factors of prolonged viable virus shedding in immunocompromised patients with COVID-19: a prospective cohort study. *Journal of Infection*. 2023;86:412-414. doi:10.1016/j.jinf.2023.01.024

4. Kim H, Yang JS, Ko JH, et al. Can nirmatrelvir/ritonavir treatment shorten the duration of COVID-19 isolation? *Front Med (Lausanne)*. 2022;9:988559. doi:10.3389/fmed.2022.988559

5. Emma Hodcroft. CoVariants. Nextstrain. Published 2023. Accessed April 14, 2023. https://covariants.org/

6. Sonnleitner ST, Prelog M, Sonnleitner S, et al. Cumulative SARS-CoV-2 mutations and corresponding changes in immunity in an immunocompromised patient indicate viral evolution within the host. *Nat Commun*. 2022;13(1):2560. doi:10.1038/s41467-022-30163-4

7. Kim C, Ryu DK, Lee J, et al. A therapeutic neutralizing antibody targeting receptor binding domain of SARS-CoV-2 spike protein. *Nat Commun*. 2021;12(1):288. doi:10.1038/s41467-020-20602-5

8. Noh JY, Yang JS, Hwang SY, et al. Duration of humoral immunity and cross-neutralizing activity against the Alpha, Beta, and Delta variants after wild-type severe acute respiratory syndrome coronavirus 2 infection: A prospective cohort study. *J Infect Dis*. 2022;226(6):975-978. doi:10.1093/infdis/jiac050

9. Cohen BJ, Audet S, Andrews N, Beeler J. Plaque reduction neutralization test for measles antibodies: Description of a standardised laboratory method for use in immunogenicity studies of aerosol vaccination. *Vaccine*. 2007;26(1):59-66. doi:https://doi.org/10.1016/j.vaccine.2007.10.046

10. Ordaya EE, Vergidis P, Razonable RR, Yao JD, Beam E. Genotypic and predicted phenotypic analysis of SARS-COV-2 Omicron subvariants in immunocompromised patients with COVID-19 following tixagevimab-cilgavimab prophylaxis. *J Clin Virol*. 2023;160:105382. doi:10.1016/j.jcv.2023.105382

11. Liu Z, VanBlargan LA, Bloyet LM, et al. Identification of SARS-CoV-2 spike mutations that attenuate monoclonal and serum antibody neutralization. *Cell Host Microbe*. 2021;29(3):477-488.e4. doi:10.1016/j.chom.2021.01.014

12. Cao Y, Yisimayi A, Jian F, et al. BA.2.12.1, BA.4 and BA.5 escape antibodies elicited by Omicron infection. *Nature*. 2022;608(7923):593-602. doi:10.1038/s41586-022-04980-y

13. Wang Q, Iketani S, Li Z, et al. Antigenic characterization of the SARS-CoV-2 Omicron subvariant BA.2.75. *Cell Host Microbe*. 2022;30(11):1512-1517.e4. doi:10.1016/j.chom.2022.09.002

14. Zhou H, Dcosta BM, Landau NR, Tada T. Resistance of SARS-CoV-2 Omicron BA.1 and BA.2 variants to vaccine-elicited sera and therapeutic monoclonal antibodies. *Viruses*. 2022;14(6):1334. doi:10.3390/v14061334

15. Motozono C, Toyoda M, Zahradnik J, et al. SARS-CoV-2 spike L452R variant evades cellular immunity and increases infectivity. *Cell Host Microbe*. 2021;29(7):1124-1136.e11. doi:10.1016/j.chom.2021.06.006

16. Lennerstrand J, Palanisamy N. Global prevalence of adaptive and prolonged infections’ mutations in the receptor-binding domain of the sars-cov-2 spike protein. *Viruses*. 2021;13(10):1974. doi:10.3390/v13101974

17. Ko KKK, Yingtaweesittikul H, Tan TT, et al. Emergence of SARS-CoV-2 spike mutations during prolonged infection in immunocompromised hosts. *Microbiol Spectr*. 2022;10(3):e0079122. doi:10.1128/spectrum.00791-22

18. Starr TN, Greaney AJ, Addetia A, et al. Prospective mapping of viral mutations that escape antibodies used to treat COVID-19. *Science (1979)*. 2021;371:850-854.

19. McCarthy KR, Rennick LJ, Nambulli S, et al. Recurrent deletions in the SARS-CoV-2 spike glycoprotein drive antibody escape. *Science (1979)*. 2021;371(6534):1139-1142.

20. Guo H, Fan Q, Song S, et al. Increased resistance of SARS-CoV-2 Lambda variant to antibody neutralization. *J Clin Virol*. 2022;150-151:105162. doi:10.1016/j.jcv.2022.105162

21. Hachmann NP, Miller J, Collier A ris Y, et al. Neutralization Escape by SARS-CoV-2 Omicron Subvariants BA.2.12.1, BA.4, and BA.5. *New England Journal of Medicine*. 2022;387(1):86-88. doi:10.1056/nejmc2206576

22. Gangavarapu K, Latif AA, Mullen JL, et al. Outbreak.info genomic reports: scalable and dynamic surveillance of SARS-CoV-2 variants and mutations. *Nat Methods*. 2023;20(4):512-522. doi:10.1038/s41592-023-01769-3

23. Tsueng G, Mullen JL, Alkuzweny M, et al. Outbreak.info Research Library: a standardized, searchable platform to discover and explore COVID-19 resources. *Nat Methods*. 2023;20(4):536-540. doi:10.1038/s41592-023-01770-w

24. Abbasian MH, Mahmanzar M, Rahimian K, et al. Global landscape of SARS-CoV-2 mutations and conserved regions. *J Transl Med*. 2023;21(1). doi:10.1186/s12967-023-03996-w

25. Badua CLDC, Baldo KAT, Medina PMB. Genomic and proteomic mutation landscapes of SARS-CoV-2. *J Med Virol*. 2021;93(3):1702-1721. doi:10.1002/jmv.26548

26. Mullick B, Magar R, Jhunjhunwala A, Barati Farimani A. Understanding mutation hotspots for the SARS-CoV-2 spike protein using Shannon Entropy and K-means clustering. *Comput Biol Med*. 2021;138:104915. doi:10.1016/j.compbiomed.2021.104915

27. Saha I, Ghosh N, Sharma N, Nandi S. Hotspot mutations in SARS-CoV-2. *Front Genet*. 2021;12:753440. doi:10.3389/fgene.2021.753440
