# Supplementary material for: Mutational analysis of severe acute respiratory syndrome coronavirus 2 in immunocompromised patients with persistent viral detection using whole genome sequencing
Source: Clin Transl Med. 2023 Nov 6;13(11):e1462. doi: 10.1002/ctm2.1462 (PMC10626488; doi:10.1002/ctm2.1462)
Supplement: Supplementary file 3 — Supporting information [file CTM2-13-e1462-s003.docx]

**TABLE S1** Clinical characteristics of the immunocompromised patients.

| **Characteristic** | **Number (%)** |
| --- | --- |
| **Age (years), mean (±SD)** | 55.8 (±9.0) |
| **Male** | 9 (69.2) |
| **Immunocompromised condition** |  |
| Hematologic malignancy | 11 (84.6) |
| Acute myelogenous leukemia | 4 (30.8) |
| Acute lymphocytic leukemia | 1 (7.7) |
| Non-Hodgkin lymphoma | 6 (46.2) |
| Autologous SCT | 1 (7.7) |
| Allogeneic SCT | 5 (38.5) |
| Solid organ transplant | 2 (15.4) |
| kidney | 1 (7.7) |
| liver | 1 (7.7) |
| B cell-depleting agent^a^ | 6 (46.2) |
| **Comorbidity** |  |
| Diabetes mellitus | 2 (15.4) |
| Hypertension | 3 (23.1) |
| Chronic kidney disease | 1 (7.7) |
| Peripheral vascular disease | 1 (7.7) |
| Cerebrovascular accident | 1 (7.7) |
| Connective tissue disease | 3 (23.1) |
| **Charlson comorbidity index, median (IQR)** | 3 (3-4) |
| **SARS-CoV-2 vaccination status** |  |
| None | 8 (61.5) |
| Partial^b^ | 3 (23.1) |
| Full^c^ | 2 (15.4) |
| **Treatment for SARS-CoV-2 infection** |  |
| Remdesivir | 13 (100.0) |
| Dexamethasone | 6 (46.2) |
| Baricitinib | 4 (30.8) |
| Tocilizumab | 3 (23.1) |
| Tixagevimab-cilgavimab | 1 (7.7) |
| Nirmatrelvir/ritonavir | 2 (15.4) |
| **Initial SARS-CoV-2 lineage** |  |
| BA.1.1 | 1 (7.7) |
| BA.2 | 5 (38.5) |
| BA.2.3 | 4 (30.8) |
| BA.2.3.11 | 1 (7.7) |
| BA.2.10 | 1 (7.7) |
| BA.5.2 | 1 (7.7) |

Abbreviations: SD, standard deviation; SCT, stem cell therapy; IQR, interquartile range; SARS-CoV-2, severe acute respiratory syndrome-coronavirus-2.

^a^ Use of anti-CD20 monoclonal antibodies or bispecific T cell engagers within two years.

^b^ One or two vaccinations against SARS-CoV-2 with Comirnaty^®^, Spikevax^®^, or Vaxzevria^®^.

^c^ More than two vaccinations against SARS-CoV-2 with Comirnaty^®^, Spikevax^®^, or Vaxzevria^®^.

**TABLE S2** Characteristics of acquired nonsynonymous SARS-CoV-2 mutations in immunocompromised patients.

| **Patient** | **Immunocompromised condition** | **Number of acquired mutations (duration of observation)** | **Mutations in S region, n (%)** | **Mutations associated with immune evasion, n (%)** | **Mutations found in major variants, n (%)** |
| --- | --- | --- | --- | --- | --- |
| A | lymphoma | 1 (28 days) | 1 (100.0) | 0 (0.0) | 1 (100.0) |
| B | AML | 8 (78 days) | 2 (25.0) | 1 (12.5) | 0 (0.0) |
| C | Kidney transplantation | 6 (33 days) | 1 (16.7) | 1 (16.7) | 2 (33.3) |
| D | Lymphoma | 28 (155 days) | 5 (17.9) | 0 (0.0) | 2 (7.1) |
| E | Lung transplantation | 11 (63 days) | 7 (63.6) | 5 (45.5) | 4 (36.4) |
| F | lymphoma | 5 (51 days) | 3 (60.0) | 1 (20.0) | 0 (0.0) |
| G | ALL | 1 (45 days) | 0 (0.0) | 0 (0.0) | 0 (0.0) |
| H | lymphoma | 15 (136 days) | 2 (13.3) | 1 (6.7) | 1 (6.7) |
| I | AML | 7 (21 days) | 5 (71.4) | 2 (28.6) | 3 (42.9) |
| J | AML | 2 (19 days) | 1 (50.0) | 1 (50.0) | 1 (50.0) |
| K | Lymphoma | 0 (13 days) | - | - | - |
| L | Lymphoma | 1 (63 days) | 1 (100.0) | 0 (0.0) | 0 (0.0) |
| M | AML | 2 (57 days) | 1 (50.0) | 0 (0.0) | 0 (0.0) |

Abbreviations: n, number; AML, acute myelogenous leukemia; ALL, acute lymphocytic leukemia.
