# Supplementary figures and images for: Mutational analysis of severe acute respiratory syndrome coronavirus 2 in immunocompromised patients with persistent viral detection using whole genome sequencing
Source: Clin Transl Med. 2023 Nov 6;13(11):e1462. doi: 10.1002/ctm2.1462 (PMC10626488; doi:10.1002/ctm2.1462)

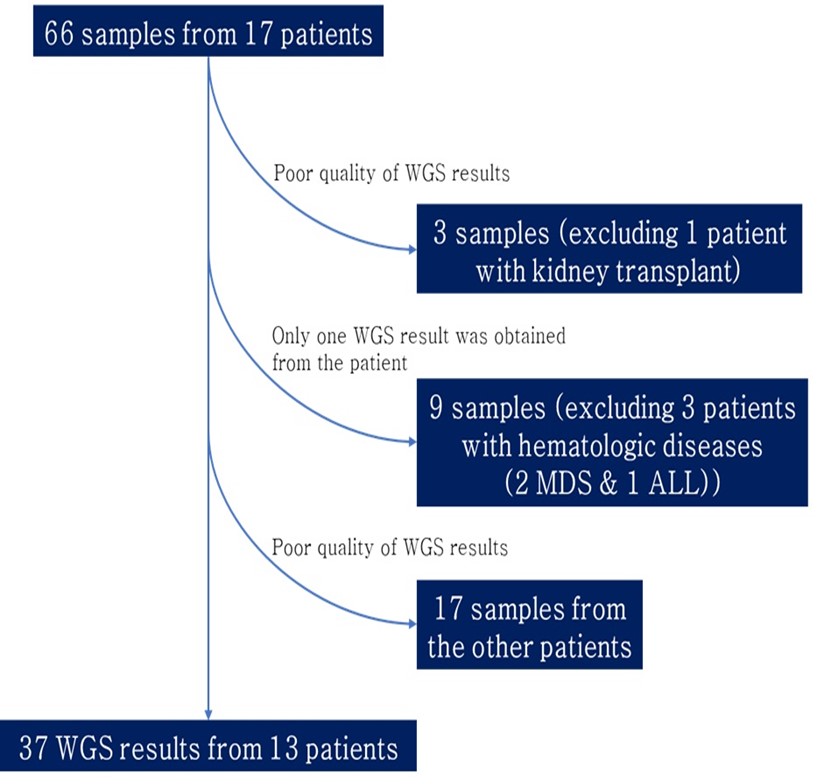

Supplement: Supplementary file 4 — Supporting information [file CTM2-13-e1462-s005.jpg]

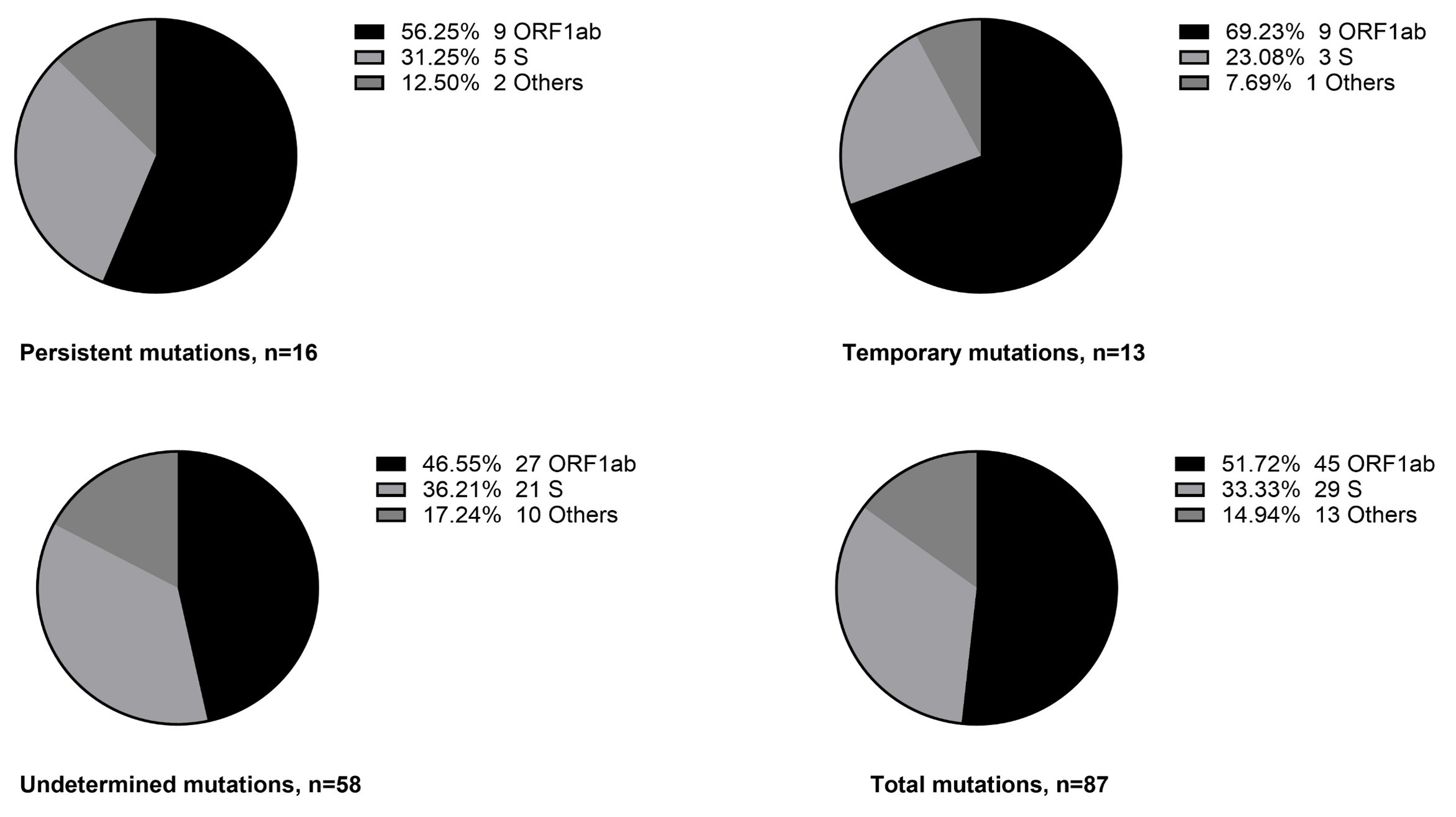

Supplement: Supplementary file 5 — Supporting information [file CTM2-13-e1462-s001.jpg]
