# Supplementary material for: Mutational analysis of severe acute respiratory syndrome coronavirus 2 in immunocompromised patients with persistent viral detection using whole genome sequencing
Source: Clin Transl Med. 2023 Nov 6;13(11):e1462. doi: 10.1002/ctm2.1462 (PMC10626488; doi:10.1002/ctm2.1462)
Supplement: Supplementary file 6 — Supporting information [file CTM2-13-e1462-s006.docx]

**Appendix Table 1** Primers and probes used in real-time RT-PCR assays to detect the N and S genes of SARS-CoV-2^a^.

| **Target**  **(Accession #)** | **Name** | **Location** | **Sequence** | **Modification** |
| --- | --- | --- | --- | --- |
| **N gene**  **(NC_045512)** | NF | 29356 | AACATTCCCACCAACAGAGC |  |
|  | NR | 29529 | GCCTGAGTTGAGTCAGCACT |  |
|  | NP | 29462 | GCTGATGAAACTCAAGCCTTACCGCA | 5’Cy5, 3’BHQ2 |
| **S gene**  **(NC_045512)** | SF | 21624 | GAACTCAATTACCCCCTGCAT |  |
|  | SR | 21787 | ACCATTGGTCCCAGAGACAT |  |
|  | SP | 21657 | TCACACGTGGTGTTTATTACCCTGACA | 5’FAM, 3’BHQ1 |
| **Internal control**  **(NC_000007.14)** | BAF | 1670 | ACTAACACTGGCTCGTGTGA |  |
|  | BAR | 1774 | CTTGGGATGGGGAGTCTGTT |  |
|  | BAP | 1700 | AGGCTGGTGTAAAGCGGCCTTGG | 5’HEX, 3’BHQ1 |

^a^ The samples were considered positive for SARS-CoV-2 genomic RNA when PCR results for both N and S genes, as well as the internal control, were positive.

**Appendix Table 2** Frequency of each mutation observed in the retrieved SARS-CoV-2 genomic sequences.

| **Mutation found in**  **BA.2 lineage** | **Number of sequences harboring the mutation among 62,219 BA.2 sequences (%)** |
| --- | --- |
| ORF1ab:I1203T | 2 (0.00) |
| ORF1ab:T1638I | 9 (0.01) |
| ORF1ab:F2122L | 27 (0.04) |
| ORF1ab:N2317D | 1 (0.00) |
| ORF1ab:A2637T | 13 (0.02) |
| ORF1ab:V2786I | 0 (0.00) |
| ORF1ab:M3733T | 6 (0.01) |
| **ORF1ab:L3829F** | 212 (0.34) |
| ORF1ab:A3969V | 4 (0.01) |
| ORF1ab:G4287R | 6 (0.01) |
| ORF1ab:T4311I | 38 (0.06) |
| ORF1ab:V4558A | 0 (0.00) |
| ORF1ab:P5360S | 287 (0.46) |
| ORF1ab:R6958K | 480 (0.77) |
| **S:HV69-70Del** | 64 (0.10) |
| **S:Y144Del** | 12 (0.02) |
| **S:K147E** | 19 (0.03) |
| **S:AL243-244Del** | 0 (0.00) |
| S:H245N | 20 (0.03) |
| **S:L368I** | 10 (0.02) |
| **S:S408R** | 206 (0.33) |
| S:V445I | 2 (0.00) |
| **S:G446D** | 2 (0.00) |
| **S:L452R** | 30 (0.05) |
| **S:Q452L** | 1 (0.00) |
| **S:V483A** | 0 (0.00) |
| **S:R493Q** | 320 (0.51) |
| **S:H505Y** | 295 (0.47) |
| **S:T547K** | 164 (0.26) |
| S:R634H | 0 (0.00) |
| ORF3:M1T | 1 (0.00) |
| ORF3:V259L | 19 (0.03) |
| M:M1T | 0 (0.00) |
| **M:D3G** | 54 (0.09) |

| **Mutation found in**  **BA.2.3 lineage** | **Number of sequences harboring the mutation among 6,006 BA.2.3 sequences (%)** |
| --- | --- |
| ORF1ab:G5063S | 4 (0.07) |
| ORF1ab:T1822I | 5 (0.08) |
| ORF1ab:D4165Y | 0 (0.00) |
| ORF1ab:R3662H | 1 (0.02) |
| ORF1ab:N4358K | 3 (0.05) |
| ORF1ab:V5184I | 0 (0.00) |
| ORF1ab:Y5223H | 0 (0.00) |
| ORF1ab:M5557I | 3 (0.05) |
| ORF1ab:S6375N | 0 (0.00) |
| ORF1ab:V6624A | 0 (0.00) |
| ORF1ab:I1505T | 1 (0.02) |
| ORF1ab:P2046S | 2 (0.03) |
| S:S255F | 9 (0.15) |
| S:Y248N | 1 (0.02) |
| **S:N405D** | 11 (0.18) |
| S:K440E | 0 (0.00) |
| **S:V445F** | 0 (0.00) |
| **S:R493Q** | 10 (0.17) |
| ORF3:I7T | 0 (0.00) |
| M:L17F | 2 (0.03) |
| ORF7b:L25F | 0 (0.00) |
| N:A35V | 0 (0.00) |

| **Mutation found in**  **BA.5.2 lineage** | **Number of sequences harboring the mutation among 656 BA.5.2 sequences (%)** |
| --- | --- |
| ORF1ab:C5191Y | 0 (0.00) |
| S:S408R | 13 (1.98) |

| **Mutation found in**  **BA.1.1 lineage** | **Number of sequences harboring the mutation among 1,721 BA.1.1 sequences (%)** |
| --- | --- |
| ORF1ab:E102K | 0 (0.00) |
| ORF1ab:I114T | 0 (0.00) |
| ORF1ab:A372V | 0 (0.00) |
| ORF1ab:V1117I | 0 (0.00) |
| ORF1ab:V1222A | 0 (0.00) |
| ORF1ab:Q1365P | 0 (0.00) |
| ORF1ab:I1367L | 0 (0.00) |
| ORF1ab:R1404C | 0 (0.00) |
| ORF1ab:A2098T | 0 (0.00) |
| ORF1ab:S2352N | 0 (0.00) |
| ORF1ab:R2695S | 0 (0.00) |
| ORF1ab:G2696A | 0 (0.00) |
| ORF1ab:D3222N | 0 (0.00) |
| ORF1ab:L3919F | 0 (0.00) |
| ORF1ab:P3952S | 1 (0.06) |
| ORF1ab:Q4100R | 0 (0.00) |
| ORF1ab:V4101L | 0 (0.00) |
| ORF1ab:A5017V | 0 (0.00) |
| **S:211-214** | Variable^*^ |
| S:R273K | 0 (0.00) |
| S:R346K | 51 (2.96) |
| S:D936Y | 0 (0.00) |
| M:Y71H | 0 (0.00) |
| ORF7:T39I | 3 (0.17) |
| ORF7:A105V | 0 (0.00) |
| N:D144H | 1 (0.06) |

^*^85-86% of BA.1.1 sequences possess the S:211-214 mutation.
